# Nest Characteristics of the Sumatran Orangutan (*Pongo abelii*) in the Wildlife Sanctuary Soraya Station in Aceh Province, Indonesia

**DOI:** 10.21315/tlsr2021.32.3.9

**Published:** 2021-09-30

**Authors:** Rita Andini, Erdiansyah Rahmi, Mardiana, Saida Rasnovi, Martunis, Ryan Moulana

**Affiliations:** 1 Faculty of Agriculture, Universiti Syiah Kuala, Jl. Teungku Hasan Krueng Kalee No. 3, Kopelma Darussalam, Banda Aceh, Aceh 23111, Indonesia; 2 Faculty of Veterinary Medicine, Universiti Syiah Kuala, Jl. Teungku Hasan Krueng Kalee No. 3, Kopelma Darussalam, Banda Aceh, Aceh 23111, Indonesia; 3 Forestry Department (Program Studi di Luar Kampus – PSDKU) Gayo Lues, Faculty of Agriculture, Universiti Syiah Kuala, Jl. Teungku Hasan Krueng Kalee No. 3, Kopelma Darussalam, Banda Aceh, Aceh 23111, Indonesia; 4 Faculty of Mathematics and Natural Sciences, Universiti Syiah Kuala, Jl. Teungku Hasan Krueng Kalee No. 3, Kopelma Darussalam, Banda Aceh, Aceh 23111, Indonesia; 5 Agricultural Processing and Technology Department, Faculty of Agriculture, Universiti Syiah Kuala, Jl. Teungku Hasan Krueng Kalee No. 3, Kopelma Darussalam, Banda Aceh, Aceh 23111, Indonesia

**Keywords:** Biodiversity, Conservation, Leuser, National Park, Protected Forest

## Abstract

Orangutans (*Pongo* spp.) populations used to be widely distributed throughout Southeast Asia, from Java in the south to the Southern China in the north during the Pleistocene. Their populations have declined up to 75% of their original size and are now distributed only in parts of the tropical rainforests of Borneo and Sumatra. *Pongo pygmaeus*, *Pongo tapanuliensis* and *Pongo abelii* are the three most representative species, in this study, here we discussed the latter. Sumatran forests are generally suffering from deforestation with rates ranging from 3.74% to 49.85% between 2000 and 2012. Thus, human wildlife conflict intensity has escalated and gained more traction. Orangutans are known as arboreal great apes and need to build nests for resting. We applied the transect line method (three transects; each 1,000 m long) at different elevations in Soraya Research Station, Gelombang Village, Sultan Daulat sub-district, Subulussalam district, and assessed the nest characteristics. The characteristics are: (1) nesting position referring to the position of nest on a tree; (2) nest successional stages defining the age and leaf decay used in constructing a nest indicated with I (new) until V (almost gone); and (3) nest density to predict the density of nest per square km. Afterwards, the identified nesting trees along the transect were further identified based on their species, and assessed based on three characteristics (the tree height, diameter and the height of a nest measured from the soil). A total of 27 nests were found, and 44% were located in transect III or at the riparian. Out of 27, four orangutans’ nests were found on Moraceae (*Streblus elongatus*) and Myrtaceae (*Syzigium* spp.), while three nests were found on *Dipterocarpus* sp. The tree height, tree diameter and nest height were 10 m–25 m (mean = 17.5 m; SD = ± 0.25), 10 cm–30 cm (mean = 20 cm; SD = ± 0.4) and 16 m–20 m (mean = 18 m; SD = ± 0.35), respectively. Meanwhile, nest density calculated based on the form: *d* = [N/ (L* 2w)], and the values obtained were 8.4, 13.45, 26.9 nests/km^2^ located on transect I, II and III or at the riparian. The most commonly found nest successional stages and position were stage III and position 3, respectively. This study could serve as a baseline research in primate conservation and nest characterisation could be used as guidance for any future activity planning (e.g. tree reforestation) in a particular region and the existence of various tree species diversity are indispensable for maintaining orangutan habitats’ quality.

**Highlights**

- Orangutans (*Pongo pygmaeus*, *Pongo tapanuliensi*, *Pongo abelii*) are the three most representative species, and here we discussed the latter. The existence of *P. abeliii* on Sumatran island is being threatened due to high deforestation rate occurred between 2000 and 2012, with a range between 3.74% up to 49.85%.
- Nesting characteristics of *Pongo abelii* in the wildlife sanctuary Soraya station in Aceh were being discussed here as nest availability could be applied as an indirect indicator of orangutan’s life quality.
- Some tree species used by orangutan for their nesting and feeding were also being highlighted here. These kind of trees should be preserved in any orangutan’s conservation efforts.

## INTRODUCTION

Orangutans (*Pongo* spp.) or the Asian Great Apes were generally found throughout Southeast Asia, from Java in the south to the Southern China in the north, during the Pleistocene or about 12,500 years ago (Chapman *et al*. 2020; van Schaik *et al*. 2001). Nowadays, their populations are declining up to 75% and distributed only in the tropical rainforests of Borneo and Sumatra (Chapman *et al*. 2020). Three prominent species known are: *Pongo abelii* on Sumatran island, *P. pygmaeus* on Borneo (Ancrenaz *et al*. 2017) and *P. tapanuliensis*. *P. pygmaeus* is phylogenetically closer to *P. tapanuliensis* although they live on separated island. *P. tapanuliensis* was recently discovered in 2018 and is being termed as “new” species (Nasution *et al*. 2018). The key determinant characteristics between the orangutan in Borneo (*P. pygmaeus*) and Sumatran orangutan (*P.abelii*) have been well described by Prayogo *et al*. (2014). Other than *P. pygmaeus*, there are still three subspecies: *Pongo pygmaeus pygmaeus*, *P. p. wurmbii* and *P. p. morio* that are still exist on Borneo island but in a very limited number (Prayogo *et al*. 2014).

The tropical rainforests serve as an essential habitat for orangutans. Sadly, the quality of their natural habitats are getting more and more deteriorated as nearly half of the remaining tropical rainforests have been already degraded and changed into fragmented forests with an area between 0.25 and 17 ha (Chapman *et al*. 2020). To amplify the current situation, the remaining degraded forests in the tropics are increasingly being converted to industrial tree plantations, for instance, for large scale oil palm expansion and establishments, deforestation for agriculture and timber extraction (van Schaik *et al*. 2001; Singh & Yan 2021). An astonishing deforestation rate of 7.2% up to 23% on average was recorded in most Southeast Asian countries, and this has been clearly driven by the increase of human population, high consumption rate and housing settlements (Chapman *et al*. 2020) and land conversion for agriculture (Supriatna *et al*. 2017). Sumatran forests are suffering from one of the highest rates of destruction in the world with a deforestation rate ranging from 3.74% to 49.85% between 2000 and 2012 or amounting to 3.55 million ha, based on what has been recorded (Supriatna *et al*. 2017). Thus, humans and primates have been trapped in a vicious conflict but primates suffered more. If no effective conservation action is taken, the orangutans (*P. abelii*) may not survive to the end of this century (Chapman *et al*. 2020).

Sumatra, with a total forest area of 2,270,080 ha, is known as the last “frontier” of wildlife sanctuaries, including orangutan, as there are two national parks (NPs): (1) the Rawa Singkil Game Reserve on the southern border and (2) Gunung Leuser NP extended to three regions, namely, Ketambe, Suaq Balimbing and Soraya, with a total area of 830.269 ha within the Aceh Province, while 205,355 ha is located in the North Sumatra Province (https://gunungleuser.or.id/kondisi-umum/, accessed on 11 August 2021). The first two regions are known as the prime habitats for orangutan and distinguished by low levels of human disturbance, high levels of tree fruit availability, and high population density of orangutan (0.43–10.18 individuals/km^2^) (Roth *et al*. 2020), whereas the latter is regarded as one of the Sumatran biodiversity hotspots close to 6,000 ha (Iqbar 2015).

Despite its pristine condition, a deforestation rate of 6.72% has occurred even within the area of protected forests, that is between Gunung Leuser NP and Batang Gadis NP in the south, with a total forest encroachment of up to 870 ha/year (KOMPAS 2019, February; Robertson & van Schaik 2001), which is likely accelerating the orangutan extinction. It is generally known that orangutans are (semi-) solitary, which means they are often found on their own but sometimes also form groups (Roth *et al*. 2020). A direct encounter is sometimes difficult, therefore, manual counting of nests from the ground, including nest characterisation [nest position referring to the position of nest on a tree (Rifai *et al*. 2013), and nest age referring the age and quality of leaves used in a nest construction (Ancrenaz *et al*. 2004)], could be applied as the simplest method to estimate orangutans’ population density (Khotiem *et al*. 2014) in Soraya Research Station. Meanwhile, tree species identification could give an overview of the environmental quality of orangutan’s habitat surrounding Soraya Research Station. Such sampling could serve as a base line research of the current status of protected forests in Sumatra.

The most important daily activities of orangutan are hanging, travelling, feeding, nest building and resting in the nest. Up to 38% of their daily time is spent for nesting, while building their nests is accounted about 5% (Rangkuti *et al*. 2015), as they generally require uninterrupted and high-quality sleep for brain and body fitness (Reinhardt 2020). Building new nests daily, intended for its rest relies on the materials’ availability such as intact and broken branches, tree rests and tender leaves derived from their surroundings. For the night rest, orangutans use their nest one time only and seldom re-use their nest. In some particular cases, they would add another new branch or leaves from their surroundings if they had a necessity to re-use it (Atmoko & Rifqi 2012). The nest successional stages indicate the age and leaf decay used in constructing a nest using these parameters: I = new (indicated with the presence of green leaves), II = recent, III = old, IV = very old, and V = almost gone (Ancrenaz *et al*. 2004) (Tables 1 and 2). The form of nests is varied, for example: as a massive ball, a densely cylindrical form, an irregular shape or a big ball shape but forming a hole on one side; with the first one as the most common one. Regardless of the form or shape, a strong structure that is safe and stable enough to attain higher levels of comfort and safety is necessary (Rifai *et al*. 2013).

This study aimed to: (1) identify the nest characteristics of orangutans living in Soraya Research Station, including nest counting, nest pattern and nest age; (2) identify tree species that were used for nesting or both (nesting and feeding); and (3) measure the quantitative and qualitative traits of the nesting tree. This information is necessary to identify orangutan’s needs in connection with the active conservation efforts in Aceh Province.

## MATERIALS AND METHODS

### Study Site

**Figure 1:**
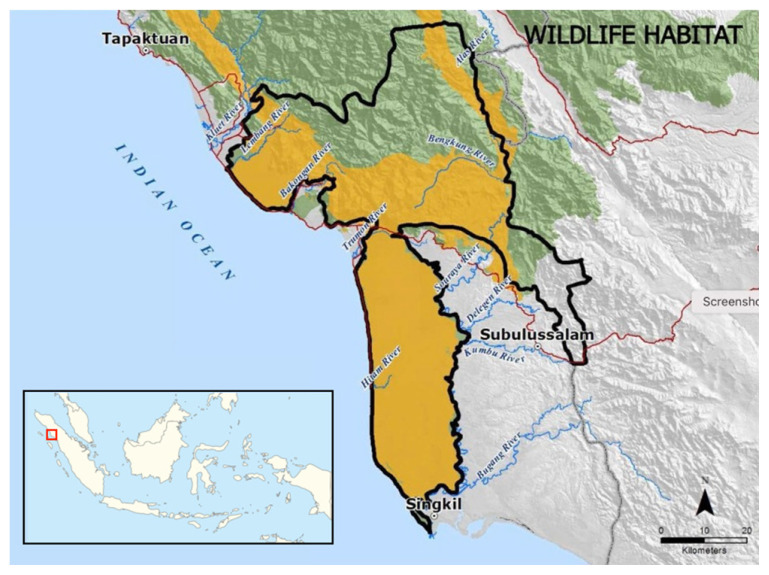
Map of Soraya Research Station in Aceh, Indonesia.

The study site is characterised by a warm, wet and humid tropical climate indicated with 88.8%–94.3% of relative humidity. The temperature range was between 21°C and 28°C with an annual precipitation of 2,450 mm (Iqbar 2015). The data was collected in Gelombang Village at Soraya Research Station (2°55′25″N, 97°55′25″E), Sultan Daulat sub-district, Subulussalam district, Aceh Province and its topography is being classified as lowland forests with an elevation ranging from 75 to 350 m above sea level (a.s.l.) (Fig. 1).

The dynamics of changes of the park management have been well described by Robertson and van Schaik (2001). The park itself is large comprising up to c. 9,000 square km and rich in biodiversity of flora and fauna: particularly its endemic birds. The vegetation inhabits the station can be classified into four zones of flora vegetation:

1. Tropical zone vegetation, which is mostly dominated by wood trees up to 40 m high.
2. Transition zone from ‘Colline’ to sub-mountainous one, where the population of wood tree species is mostly replaced with various colourful flower species that are already adapted in various altitudes and the ‘spiny’ *Calamus* spp.
3. Montane areas, which is dominated by 10 m–20 m trees and covers a small proportion of prime habitats for Sumatran endemic mammals.
4. Sub-alphine zone (https://www.conservationatlas.org/gunung-leuser-national-park).

Initially, the Soraya conservation park was once under the management of a private company, which owned the logging concessions before 1990s. In the early 1990s, the park received insufficient protection because of insufficient design of the original protected area and some deficiencies in land use planning (Robertson & van Schaik 2001). In 1994, the station was built by the Management Unit of Gunung Leuser National Park Foundation (https://www.sumatra-ecotravel.com/about-us/where-we-are/gunung-leuser-national-park/) and it has been serving as a hub for primate research, especially the Sumatran orangutan. In 2000, the original station was destroyed during the military conflict in Aceh (Utama 2019), and it was burnt in 2011. From 2011 until 2015, it was on ‘hiatus’. Since 2015, the Forum Konservasi Leuser (FKL) with other multiple parties, had rebuilt the station and continued to promote the option to conduct research to local, national and international researchers (https://leuserconservancy.or.id/).

### Data Collection

**Figure 2:**
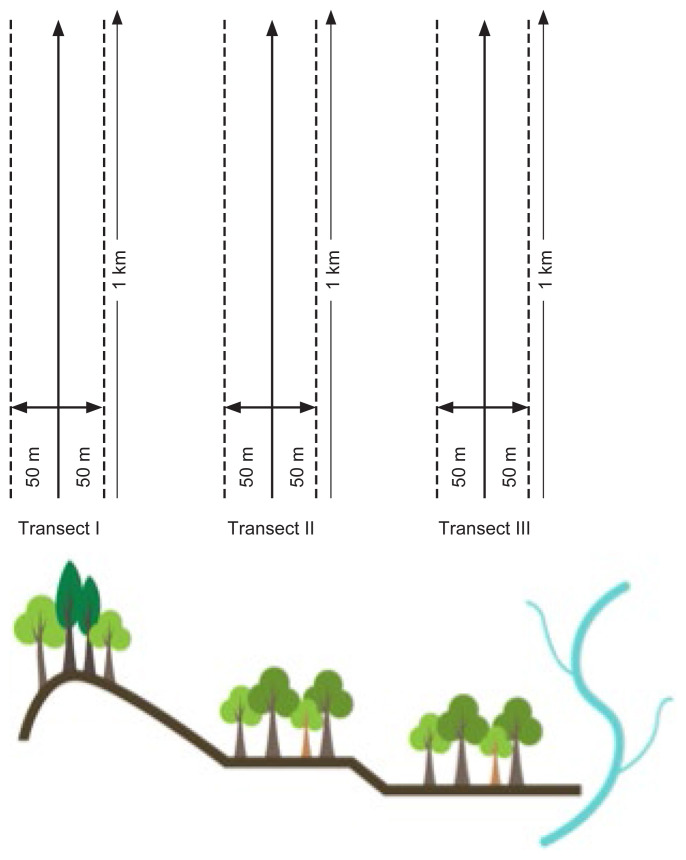
The line transect method applied in this research.

Manual nest counting from the ground using line transects was applied in order to enable us to estimate the orangutan’s nest density (Riyadi *et al*. 2015). The study was conducted in June–July 2019. The line transects were divided into three zones (*n* = 3): lowland forests with an elevation of 200 m–350 m a.s.l. (transect I), lowland forests with an elevation of 100 m–200 m a.s.l. (transect II), and riparian areas or at the riverside at 75 m–100 m a.s.l. (transect III). Each line transect established was 1,000 m long and 1 m wide (Fig. 2 and Appendix A: picture of the tract). The transect design used systematic random sampling (Nasution *et al*. 2018) based on ArcGIS 10.1. Within each line, the borders were tagged with plastic thread in red colour and data listed in Table 1 were collected.

The basic equation for nesting density derived from line transect survey is *d* = *N*/2w*L (Sidiq et al. 2015), where *d* is the nest density (nest/km^2^) briefly assumed or translated into orangutan’s density (individual/ km^2^), *N* is the number of nests observed along the transect, *w* is the perpendicular distance from the nest to the transect line and *L* = length of transect covered (km).

Abiotic condition: Average temperature and humidity data from the morning (08:30–09:00) and afternoon (15:00–16:00) were recorded by using a thermohygrometer (TL8036, China).

### Data Analysis

The data especially the nesting density and produced graphs were calculated using Microsoft Excel.

## RESULTS

The total of nest manual counting were 27, 44% of which were found in the lowlands. Meanwhile, 37% and 19% of the nests were found at the riverside and the highlands, respectively. Moreover, a total of 13 tree species were used for the majority of nests, 84.6% of which had a double function, both for nesting and for feeding. Tree species belonging to Myrtaceae (*Syzigium* spp.) with 18.5% and Moraceae (*Streblus elongatus*) with 14.8% were mostly used for nests, whereas the third most favoured species were those belongs to the family of Dipterocarpaceae, mostly represented by the Dipterocarpus sp. Next come *Cyathocalyx sumatranus*, *Sterculia* sp., *Litsea* sp., *Lophopetalum javanicum* and *Eudgenia clavimyrtus*, which belong to Annonaceae, Sterculiaceae and Lauraceae (see Table 3). These tree species are also used for nests but not during the fruit season.

In this study, we found that nesting trees with a height of 10 m to 25 m (mean = 17.5 m; SD= ± 0.25) and a diameter ranging from 10 cm to 30 cm (mean = 20 cm, SD = ± 0.4) were most commonly chosen by the orangutans living in Soraya to build their nests. The common height of the nest was 18 m high (SD = ± 0.35). Higher trees with height between 20 m–30 m with a tree diameter range between 10 cm–30 cm were more favoured by the Tapanuli orangutans (*P. tapanuliensis*) in Batang Toru regions in North Sumatra (Nasution *et al*. 2018); meanwhile, *the Borneon* orangutans (*P. p. pygmaeus*) inhabiting the West Kalimantan prefer to choose tree up to 39 m high and tree diameter of 13 cm–42 cm and in some cases up to 63 cm (Khotiem *et al*. 2014, Riyadi *et al*. 2015).

Nest characteristics consist of three components: Successional stage, position and density. In general, successional stage is closely associated with the nest quality and is divided into five categories (Table 2). At Soraya Research Station, stages III and IV, or the nests that are already more than 2 and 3 weeks old, respectively, were more common (see Table 3). The occurrence of new and fresh nests (stage I) was rare and only found on a *Dipterocarpus* tree in transect III.

The values of nest density in this result were 8.4, 13.45 and 26.9 nests/km^2^ obtained from transects I, II and III, respectively (Appendix A); with the highest number of density was found in transect III or at the riverside (75 m–100 m a.s.l.). We had encountered 5 to 10 individuals in Soraya Research Station. Meanwhile, the most commonly found nest position (up to 48%) in Soraya Research Station was position 3 [Table 2; Figs. 4(c) and 5], that seemed to be more robust compared to positions 1 and 2. This kind of nest position was in the upper parts of tree crown at sites that provided a clear view of the surroundings, although such position does not prevent strong wind and rain for the orangutans. Position 2 was the second most commonly found one with 37% [Table 2; Figs. 4 (b) and 5]. The nest at position 2 was located at the side of the first main branch without being attached to it. There was no record of nest in position 0, 1 and 4 observed in this study.

## DISCUSSION

The forests in Sumatra can still be presumed as the ‘last frontier’, or supporting habitat of orangutans, although its deforestation pace is the second highest after Borneo. In Sumatra, the majority number of orangutan can be found in Aceh Province. In the rugged mountains of Aceh, 6,600 orangutans are estimated, and they inhabit inside and outside the NPs; with the remaining 0.09% exists in West Mount Leuser (Wich *et al*. 2008). Most of the areas in NPs consist of high mountains, although their densities decrease with an increasing elevation. The distributions of orangutans were majorly found on lowlands area with an elevation below 900 m with a density of 0.30–10.80 individuals per square km (Nasution *et al*. 2018). Their populations are mostly distributed on fragmented lowland forests and peatlands less than 17 ha. To exacerbate the situation, an increase of fragmented forests up to 33-fold within the next 50 years was also being prognosed (Chapman *et al*. 2020).

Orangutan’s population density and habitats both on Sumatra and Borneo would be at an alarming rate due to such fragmented forests and ongoing deforestations (Wich *et al*. 2016). In Borneo, almost half of its original forests that were mapped in 1970s were already disappeared and had been converted to other types of land-use intended for large scale monoculture agribusiness (Ancrenaz *et al*. 2017). A similar grim situation regarding the forests condition on Sumatra was also being portrayed. In 1950, the island of Sumatra was still densely covered with big tropical trees, but nowadays, it already lost almost 70% of its forest canopy. Many factors have led to such conditions. Two major ‘root’ causes were identified: weak compliance with regulations and weak law enforcement (Robertson & van Schaik 2001). Since early 1970s, most orangutans’ populations in Sumatra and Borneo have been in a constantly dangerous condition (Supriatna *et al*. 2017). For example, the ‘rugged’ mountainous regions of Ulu Masen in Aceh and its surroundings – is known as one of the prime habitats in the northern Aceh – up to 800 individuals were estimated in the population but suprisingly, no orangutan revealed in the field survey in 2007 (Wich *et al*. 2008). Such condition on the field would fit with a future prediction made by Wich *et al*. (2008) in Aceh-only scenario. There will be a steep decline up to 68.5% of the current numbers as a result of annual forest loss up to 1.0%–1.5% in most of orangutans’ habitat in over 16 years. Meanwhile, a quite high prediction of 94.3% of the current numbers is prognosed solely for the Leuser ecosystem. Most of the scientists agreed that 250 individuals are the minimum number for a viable population. Currently, only three areas on the northern part of Sumatra have more than 1,000 individuals with another three additionals remained containing 250 until 1,000 individuals (van Schaik *et al*. 2001).

A healthy population in Leuser is ‘vital’, particularly, for the survival in the wild of the Sumatran orangutans (van Schaik *et al*. 2001). A high quality or intact forest; particularly high levels of fruit trees availability can be used as an indication of orangutan’s habitat quality. In Leuser, orangutan’s density between 0.5 until 7 individuals/km^2^ was reported, with the higher densities are the river valleys or also referred as ‘flood plains’. Our results were in line with the previous study where the tropical lowland forests were favoured for building nests (Result section: 44% of the total counted nests). Although our result did not calculate and present the actual individual density, we confirmed that a higher occurrence of nest density (28.6 nests/km^2^) at the riparian or the river valley; with an assumption of 1.8 constructed nests/day as a mean value, it could be inferred that most orangutan ‘transit’ and ‘stay’ at the riverside (Wich *et al*. 2016).

The nest quality or nest successional stage are more or less the same between Soraya park and Batang Toru, aging over two weeks old or stage III. Contrastingly, a quite low number of fresh nests was reported for the Borneo (Khotiem *et al*. 2014). This could be inferred that degradation of orangutans’ prime habitats in Borneo is deliberately more severe than in Sumatra as this was the case in the degraded forests of Kinabatangan (Malaysian Borneo). In the degraded forests, most of the resting day periods were spent on simple, unwoven mattresses of leaves, composed of the abundant leafy material of entangled vine and creepers inside the crown of most trees but most of them were poorly constructed. This suggested that they were used for rest stops between two consecutive feeding bouts, as documented for chimpanzees (Ancrenaz *et al*. 2004).

Despite an annual decrease of forest loss up to 0.6% within the Leuser ecosystem in Sumatra (Wich *et al*. 2008), it seemed that the Soraya conservation park still harbours wide range of diversity in terms of various tree species; serving for nesting and feeding. It is worth to stress that such relatively high biodiversity pronounced in Soraya conservation park could be a result of the political situation (the Aceh freedom movement) in Aceh from early 1980s to 2004, when there was hardly any significant reduction on tree biodiversity. A diverse richness of 178 tree species are reported, and 90 of it have a potential to be utilised as fruit trees or other than fruits (young leaves, flower seeds and seedlings) up to 60% as their major feed (Fauzi *et al*. 2017) in the northern part of Sumatra (Rifai *et al*. 2013).

**Figure 3:**
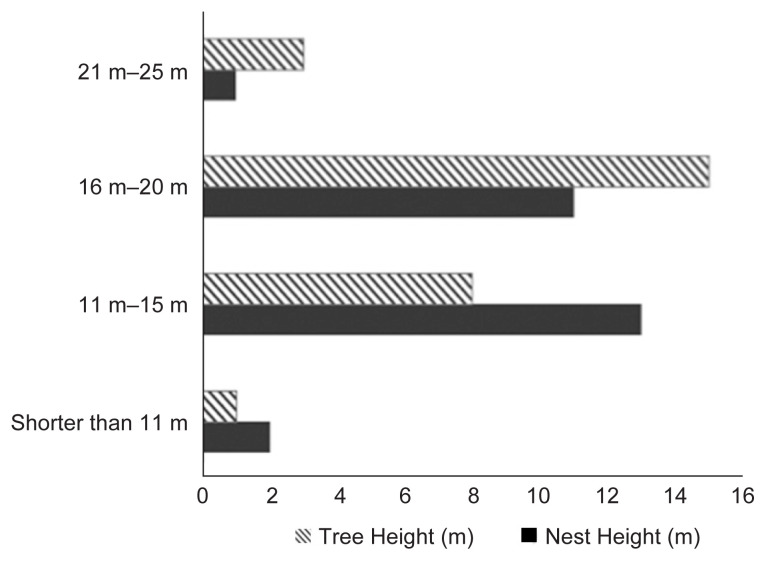
Tree height and nest height.

**Figure 4:**
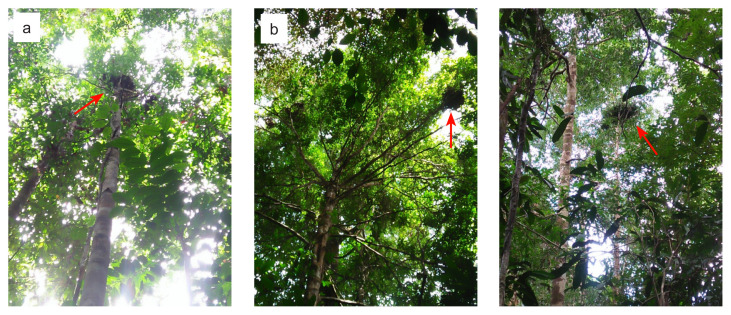
Nest position: (a) first, (b) second and (c) third (marked with red arrow).

**Figure 5:**
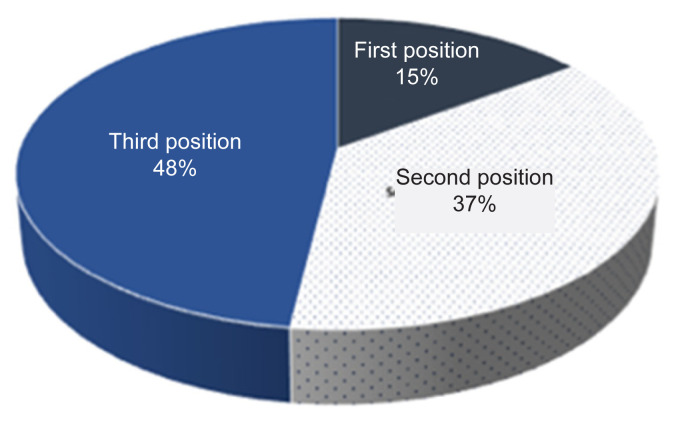
Percentage of each nest position (field observation at Soraya Research Station in 2019).

The findings confirmed that the strong and high-quality trees with larger tree diameter, such as Dipterocarpaceae or *Shorea* sp., with many branches and tender leaves were useful for building nests. Other tree species, e.g., *Agathis* sp., Anacardiaceae, *Cinnamomum* sp., Euphorbiaceae, Fagaceae, Lauraceae, Myrtaceae and Sapotaceae are also used by orangutans as nesting trees. Most of nesting trees found in Soraya conservation park were 16–20 m high with diameter of 10–25 cm. This result is in accordance with previous findings (Khotiem *et al*. 2014; Riyadi *et al*. 2015) that orangutan preferred trees with more that 15 m in height, and their nests were usually located at 13 m or above 15 m from the ground (see: Fig. 3). Orangutans favour the largest and tallest trees available in order to protect them from any natural and nocturnal predators, e.g., big phythons, tigers and leopards that could prey on younger individuals (Ancrenaz *et al*. 2004).

Meanwhile, ‘rose apple’ (*Syzygium* sp)., and ‘damli’ (*Streblus elongatus*) had a ‘double’ function. Fruit abundance was more important than tree diameter, as long as the selected trees were still able to support the body weight. High levels of fruit trees’ availability; particularly the production of fruits with soft pulp, could also be applied as an ‘indirect’ good predictor of orangutan’s density. This kind of supporting environmental factor may also explain why the variation in the population density in Sumatra was still higher than the variation in Borneo (Rifai *et al*. 2013).

Seven recommendations regarding the conservation efforts of orangutans in general in Sumatra and Borneo have been published in order to reduce hunting and human-orangutans conflict in agricultural areas; with a strong emphasise being highlighted: effective law enforcement and prosecution is needed to stop hunting of orangutans for food and trade purposes. All efforts are no avail unless the decline in numbers of orangutan is halted and at this stage, a strong political willingness; both at the national and provincial level is highly demanded (Wich *et al*. 2008). This publication would also give a ‘halt’ for the government to re-consider the ‘already’ planned new road construction (Ladia Galaska) connecting Blangkejeren to Kutacane in the Southeast Sumatra.

Orangutans are referred to as the ‘umbrella’ species because their existence could serve as a healthy indicator for protected forests and natural conservation areas. The World Bank reports based on previous monitoring activities in Africa and South America showed that the improved road-access and facilities that facilitates natural habitat degradations, particularly within the NP and its peripheries, and also the Soraya Park (Arcus Foundation 2018). A sign of ‘political changing will’ was prepared by the Ministry of Forestry, with contributions from a few renowned orangutans’ experts. Another sign of ‘political changing will’ and action have been campaigned by the local government dedicated in the current moratorium on logging in Aceh province. Other mechanisms of ‘new incentives’ for forest protection launched by the government or NGO’s have also been implemented. In combination with the above-mentioned recommendations, these may act as a ‘new’ breeze to improve the forest and orangutan protection (Wich et al. 2008).

## CONCLUSION

The three species of orangutans (*P. pygmaeus, P. abelii* and *P. tapanuliensis*) are now endangered as their populations are decreasing tremendously, at the rate up to 75% because of rapid deforestation in two Indonesian main islands: Borneo and Sumatra. Sumatran forests are also facing the deforestation rates ranging from 3.74% to 49.85% between 2000 and 2012 or equals to 3.55 million ha. This study reported the nest characteristics (nest position, successional stages and density) and the results have been explained. Orangutans are known as umbrella species related to healthy forest indicator. Therefore, their nest characterisation could be used as simple and indirect guideline in orangutan conservation efforts, particularly the existence of various tree species diversity is indispensable for maintaining orangutan habitats’ quality. Other additional information such as tree height, tree diameter and nest height were also added. We found that 85% of tree species performed a double function, both for nesting and for feeding, e.g., *Syzigium* sp. and *Streblus elongatus*.

## Figures and Tables

**Table 1:** Collected data of orangutans’ nest.

No.	Parameters	International unit	How to measure	Remarks
1	Height of the nest from the ground ⊗	metre (m)	Laser rangefinder was applied in order to measure the height of nest	
2	Nesting site (see Table 2) Ω	-	Field observation and then cross-checked with reference	0, 1, 2, 3, 4
3	Nest successional stages (see further in Table 2) Ω	-	Field observation and then cross-checked with reference*	Stages I = new until V = almost gone; further information see: Table 2
4	Nesting tree species	Nesting tree species	Nesting tree species	Nesting tree species
5	Number of nests ⊗	-	Based on the manual counting during field observation	Unit
6	Nesting density ⊗	nest/km square	Based on the manual counting data in terms of number of existing nests (N), the length of transect (L), the distance from nest to (each) transect (w) in km and then put in the form	Form: *d* = [N/ (L*2w)]
7	Nesting tree diameter ⊗	metre (m)	The diameter of tree was measured at a breast height; with a band tape	
8	Nesting tree height ⊗	metre (m)	Laser rangefinder was applied in order to measure the height of nest, then, trees were divided into four categories ^δ^	^δ^ categories: trees shorter than 11 m; trees that are 11 m–15 m tall; trees that are 16 m–20 m tall; trees that are 21 m–25 m tall

*Note*:

* Reference: Ancrenaz *et al*. (2004); type of data:

Ω = qualitative,

⊗ = quantitative

**Table 2:** Orangutans’ nest pattern and stage (Rifai *et al*. 2013).

Nest position	Position	Criteria	Nest successional stage	Leaves illustration	Nest quality	Criteria
0	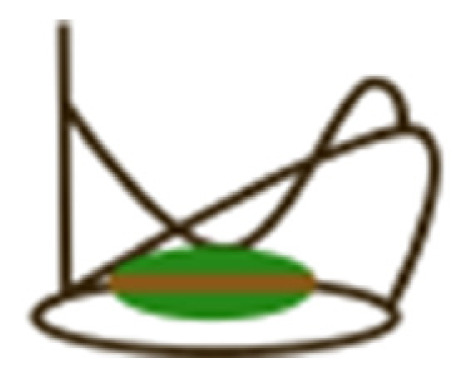	Nest was found on the soil ground. This is a very rare position found among the Sumatran orangutans; however, this is common among the Borneo orangutan.	I	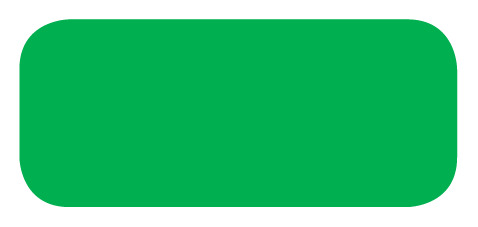	New and fresh	Green leaves are still fresh. Nest is just one day old but abandoned.
1	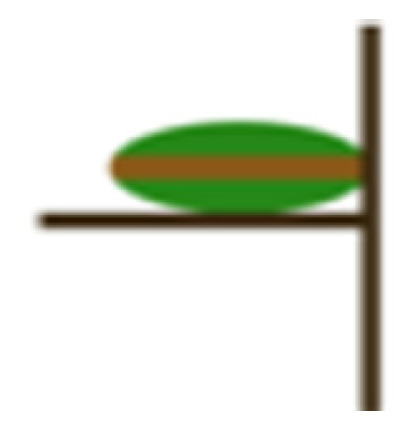	Nest was found at the first adjunction between the first (branch (left or right) and the main bark. Nest was found at the middle or at the top-edge of the first branching. Location could be at the left or right side.	II	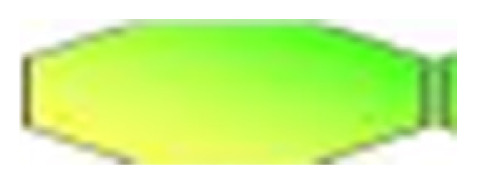	Recent	Approximately 70% – 80% of total leaves are still available but colour starts to be brown. Usually ± 2 weeks old.
2	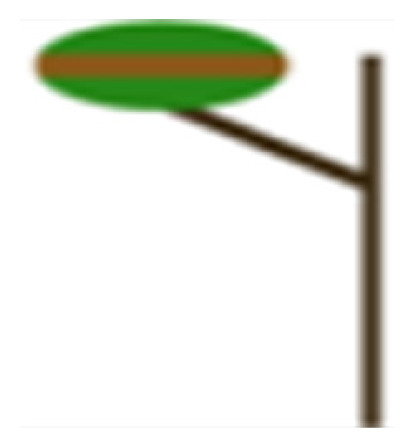		III	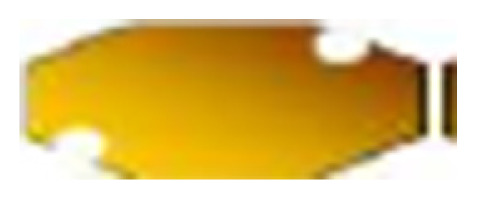	Old	Half of total leaves already disappeared, though the basic construction made of tree branches still remain firm. Usually ± 2 weeks old.
3	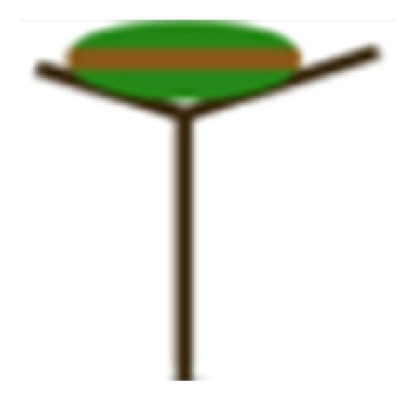	Nest was found at the branch intersection of the main bark.	IV	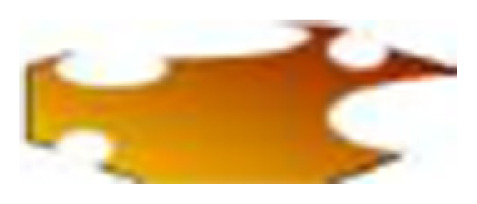	Quite old	A maximum of 30% leaves were left compared to the initial one. Most of the leaves are damaged (have holes). Usually ± 3 weeks old.
4	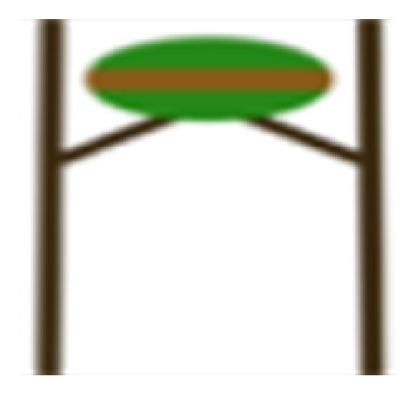	Nest was formed by adjusting more than two trees, and located at the adjunction parts.	V	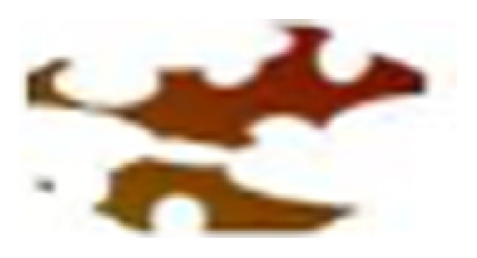	Diminishing	The basic construction has already diminished; only some tree branches and small woods were left. Usually more than 4 weeks old.

**Table 3:** Species of tropical trees used as nest and feed recorded in this study.

No.	Local name	Latin name	Family	No. of nests	Emphasis of use
1	Aging	*Renorea sclerucarpa*	Violaceae	2	Non-feed
2	Bau langit	*Cyathocalyx sumatranus*	Annonaceae	1	Feed (fruits)
3	Bulu ayam	*Sterculia* sp.	Sterculiaceae	1	Feed (fruits)
4	Damli	*Streblus elongatus*	Moraceae	4	Feed (fruits)
5	Jerik batu	*Clausena engleri*	Rutaceae	2	Feed (fruits)
6	Kayu jambu	*Syzigium* spp.	Myrtaceae	4	Feed (fruits)
7	Medang telor	*Litsea* sp.	Lauraceae	1	Feed (fruits)
8	Meranti petimah	*Lophopetalum javanicum*	Dipterocarpaceae	1	Feed (fruits)
9	Pepening	*Dipterocarpus* sp.	Dipterocarpaceae	3	Feed (fruits)
10	Rumpirawan	*Mallotus sphaerocarpus*	Euphobiaceae	2	Feed (fruits)
11	Semantok	*Eugenia clavimyrtus* K. et V	Myrtaceae	1	Non-feed
12	Setur padi	*Aglaia korthalsii*	Meliaceae	2	Feed (fruits)
13	Tampu tapak-gajah	*Macaranga triloba*	Euphorbiaceae	3	Feed (fruits)

Total				27	
